# PD07 Can Commencing Colorectal Cancer Screening At Age 45 Years Be A Good Investment? An Individual-Level Simulation Analysis In Germany

**DOI:** 10.1017/S0266462324002769

**Published:** 2025-01-07

**Authors:** Min Wai Lwin, Chih-Yuan Cheng, Silvia Calderazzo, Christoph Schramm, Michael Schlander

## Abstract

**Introduction:**

The effectiveness and cost saving advantages of colorectal cancer (CRC) screening have gained widespread scientific consensus. However, the rising incidence of early-onset CRC has challenged Germany’s current screening program, which focuses on individuals aged 50 years or older. This study evaluated the potential cost effectiveness of initiating CRC screening in Germany at the age of 45 years.

**Methods:**

The cost-effectiveness analysis utilized a validated discrete-event-simulation model, DECAS, which incorporates both adenomatous and serrated polyp pathways in CRC development. This model has been validated using German CRC epidemiological data and simulates the effects of screening interventions. It was used to compare four new CRC screening strategies starting at age 45 years (10-yearly colonoscopy, annual or biennial fecal immunochemical testing [FIT], or both) with the current screening strategy starting at age 50 years. The simulation, assuming perfect adherence, included a cohort of 100,000 individuals with an average CRC risk from age 20 to 90 years or death, applying a three percent discount with costs in 2023 Euros.

**Results:**

The model outcomes included quality-adjusted life-years (QALYs) gained and total incremental costs, considering both CRC treatment and screening costs. Initiating 10-yearly colonoscopy only or FIT plus colonoscopy strategies at age 45 years yielded incremental gains of seven to 28 QALYs, with incremental costs of EUR28,360 to EUR71,759 per 1,000 individuals, compared with the current strategy. The incremental cost-effectiveness ratios varied between EUR1,029 and EUR9,763 per QALY gained. The FIT-only strategy was dominated by the current screening strategy. These findings remained consistent throughout the probabilistic sensitivity analyses.

**Conclusions:**

The cost-effectiveness findings support initiating CRC screening at age 45 years with either colonoscopy alone or colonoscopy plus FIT, demonstrating substantial gains in QALYs and a modest increase in costs. Our findings emphasize the importance of implementing CRC screening five years earlier than the current practice to achieve more significant health and economic benefits.

